# Complete Draft Genome Sequence of *Escherichia coli* JF733

**DOI:** 10.1128/genomeA.00298-16

**Published:** 2016-04-21

**Authors:** Gabriele R. M. Kleiner, Daniel Wibberg, Anika Winkler, Jörn Kalinowski, John E. Wertz, Karl Friehs

**Affiliations:** a Fermentation Engineering, Bielefeld University, Bielefeld, Germany; b Center for Biotechnology, Bielefeld University, Bielefeld, Germany; c Department of Molecular, Cellular and Developmental Biology, E. coli Genetic Stock Center, Yale University, New Haven, Connecticut, USA

## Abstract

*Escherichia coli* JF733 is a strain with a long history in research on membrane proteins and processes. However, tracing back the strain development raises some questions concerning the correct genotype of JF733. Here, we present the complete draft genome of *E. coli* JF733 in order to resolve any remaining uncertainties.

## GENOME ANNOUNCEMENT

*Escherichia coli* JF733 (CGSC #6047) is obtainable from the E. coli Genetic Stock Center (CGSC, New Haven, CT, USA). The strain has a long history, and CGSC tries to collect and update all information about their available strains and their characteristics. Sequencing of *E. coli* JF733 enables the clarification of remaining uncertainties and gives new insight into the genetic background of *E. coli* JF733.

*E. coli* JF733 was created based on the parental strain *E. coli* AT3143 (CGSC #4539) (1, 2). The latter is an *E. coli* K-12 derivate that emerged from mating AT3055, also known as *E. coli* W1485 derivative X961, with Hfr 3000 (also AB259) (personal communication between A. L. Taylor and the CGSC). By transduction, *purF30* and *serC53* alleles were moved out, while *aroA357* was moved into the genome of *E. coli* AT3143, resulting in *E. coli* JF568 (CGSC #6041). In the next step, *aroA357* was again transduced out in order to move in *ompF254* (formerly *ompA254*), which led to *E. coli* JF699 (CGSC #6043) (personal communication between J. Foulds and the CGSC). *E. coli* JF733 was then developed by spontaneous mutations that resulted in the lack of proteins Ib and II* (2). The following JF733 genotype is currently published by the CGSC (cgsc.biology.yale.edu): [F^-^, *lacY29, proC24, tsx-63, purE41, λ^−^*, ompA252, his-53, ompC262, rpsL97(strR), xyl-14, metB65, cycA1, ilv-277, cycB2?], 2016-01-27.

Initial studies using *E. coli* JF733 were focused on functional studies of outer membrane proteins and porins (3–5) as well as on their contribution to bacteriophage and colicin sensitivity (2, 6). Furthermore, production and secretion of recombinant proteins like anti-*α*TF using JF733 were described in 2008 (7).

To resolve remaining uncertainties concerning the JF733 genotype for further research, the draft genome sequence of *E. coli* JF733 was established on the Illumina MiSeq system as recently described for other microorganisms (8–10). A paired-end sequencing run (2 × 300-bp) yielded 2,266,996 reads with a total size of 646.20 Mb. Assembly using the GS *de novo* assembler version 2.8 resulted in 107 contigs and 58 scaffolds for the JF733 draft genome. Annotation of the genome was accomplished within the GenDB platform (11). The chromosome has a size of 4,518,620 bp with a G+C content of 50.78%. In total, 4,218 coding sequences, 70 tRNA genes, and 3 species of rRNA genes were identified by the gene and RNA prediction tools.

Sequencing of the *E. coli* JF733 genome and comparison to *E. coli* W3110 (GenBank: AP009048.1) confirmed most of the indicated mutations and resulted in a specification of three affected genes, namely, *hisD*, *ilvD*, and *xylR.* No mutations in *ompC*, *metB* (and *metC*), and *cycB* were detected. Whereas the latter is not annotated for *E. coli* W3110, a potential deletion within *tsx* could not be verified with certainty using the obtained data*.* Although no mutation within *ompC* was detected, a base-pair substitution located in the −10 promoter region 90 bp upstream of *ompC* (12) was found, which could explain the described lack of protein Ib (2).

### Nucleotide sequence accession numbers.

The *E. coli* JF733 draft genome sequence was deposited in the EMBL database under the accession numbers FBSE01000001 to FBSE01000058.
